# Two-Color Fluorescence Thermometry Using Lock-in Amplifiers for Background Suppression

**DOI:** 10.3390/s25206364

**Published:** 2025-10-15

**Authors:** Benjamin R. Anderson, Hergen Eilers

**Affiliations:** 1 Applied Sciences Laboratory, Institute for Shock Physics, Washington State University, Spokane, WA 99210-1495, USA; 2 ExMat Research, Spokane, WA 99223, USA; hergen.eilers@exmatresearch.com

**Keywords:** thermometry, ratio thermometry, fluorescence, lock-in amplifiers, explosions, background suppression

## Abstract

We present a comprehensive theoretical and experimental study of the lock-in amplifier detection technique applied to two-color fluorescence thermometry. We modeled and measured the impact of different lock-in amplifier parameters (reference frequency, time constant, roll-off) and background pulse characteristics (width and steepness) on the lock-in amplifier’s background suppression, as well as modeled the impact of the lock-in amplifier parameters on temperature measurements of short duration heating events. Based on our results, we provide a guide for designing experiments using lock-in amplifiers in two-color fluorescence thermometry to obtain the best possible results.

## 1. Introduction

Two-color fluorescence thermometry is a widely used temperature sensing technique that utilizes a wide variety of thermographic phosphors (e.g., Dy:YAG [1,2,3,4], Eu:BAM [5,6], Dy/Sm-based organic complexes [7,8,9,10], Dy/Eu-doped crystalline hosts [11,12,13,14,15,16]), to measure temperature by comparing the ratio of fluorescence intensities from two closely spaced excited states, with the states having an energy level spacing of ΔE . For these states, the ratio of their intensities is related to the ratio of their populations, which obeys a Boltzmann distribution:

(1) $$\begin{array}{c} \frac{I_{2}}{I_{1}}∼\frac{n_{2}}{n_{1}}=e^{-\Delta E/kT}, \end{array}$$

(2) $$\begin{array}{c} \frac{I_{2}}{I_{1}}=Ae^{-\Delta E/kT}, \end{array}$$

where $I_{i}$ is the intensity of the *i*th energy level, $n_{i}$ is the population of the *i*th energy level, *k* is Boltzmann’s constant, and *T* is the temperature. This technique has been applied to temperature measurements involving gases [2,17,18] and surfaces [1,19,20,21] in a wide range of applications including fighter jet engines [22,23,24,25], heated jets [5], porous media burners [26], diesel engines, [27] turbine engines [28,29,30,31], combustion environments [32,33,34,35], and explosive fireballs [36,37].

In most of these applications—especially that of explosions—there is a significant amount of background light that can interfere with the fluorescence ratio calculation leading to erroneous results. Therefore, it is necessary to have techniques to account for this background light, such that the fluorescence signal can be isolated. The most direct option is to have a second detection system that only measures the background light, such that its signal can be subtracted from the combined background and fluorescence signal, but this option is not always feasible due to size, complexity, and cost restrictions. Also, it is not always guaranteed that the two detection systems see the same background light, which can lead to errors when performing the background correction.

Alternatively, for background light that is not too intense, it is possible to use a single time-gated detector and pulsed excitation to measure the fluorescence intensity on such short timescales that the background light is negligible compared to the fluorescence. For instance, we previously used this technique to measure the fluorescence spectrum of Dy:YAG up to 2033 K during laser heating by using a gate width of 300 μs [36]. As long as the background light is significantly dimmer than the fluorescence on the timescale of the gate pulse, this technique works well. Therefore, the applicability of this technique depends on the fluorescence lifetime of the thermographic phosphor and the relative intensity of the fluorescence and background light on this timescale.

While time-gated detection works in certain cases, there are many other scenarios—such as explosive fireballs—where this technique is inappropriate as the background light can be several orders of magnitude brighter than the fluorescence intensity, even on the timescale of one lifetime. This challenge is further complicated in explosive fireballs as they have light curves that fluctuate on the order of milliseconds, such that the background can vary significantly during a single relaxation [37].

For these extreme situations, we propose the use of a lock-in amplifier-based technique to remove background light. In this technique a pulsed laser (with repetition rate $\omega_{0}$ ) modulates the fluorescence from a thermographic phosphor, which is then detected by two band-pass-filtered photodetectors along with the unmodulated background. The electronic signal from each photodetector is then passed through a lock-in amplifier, which suppresses the unmodulated background and extracts the modulated fluorescence signal for each detector. The resulting lock-in output traces are then used to compute the intensity ratio trace, from which the temperature is determined.

While lock-in amplifiers are ubiquitous lab equipment and have been used extensively for fluorescence lifetime-based temperature sensing [38,39,40,41,42,43,44], there are very few papers in the literature on their application to two-color fluorescence thermometry [45]. In this paper, we seek to address this deficit with a comprehensive study of the functionality and performance of the lock-in amplifier technique applied to two-color fluorescence thermometry using both modeling and experimental measurements. We consider the impact of the different lock-in parameters (reference frequency, time constant, roll-off) as well as characteristics of the background light (pulse width and steepness) on the suppression of background light. In addition, we model the impact of the lock-in amplifier technique on the measured temperature curves. Note that for this study we primarily focus on timescales of 10’s of microseconds to milliseconds—which are the regime of interest for explosive fireballs—but the conclusions of this study can be extended and applied to other timescales as well.

## 2. Method

### 2.1. Modeling

To begin, we provide a brief overview of the functionality of a lock-in amplifier. We begin by writing the input signal, v(t) , as containing two components:

(3) $$v\left(t\right)=a\left(t\right)\sin\left(\omega_{0}t+\phi_{s}\right)+b\left(t\right),$$

where b(t) is a time varying background/noise component, a(t) is the component we want to measure that is modulated at $\omega_{0}$ and is slowly varying relative to $\omega_{0}$ , and $\sin(\omega_{0}t+\phi_{s})$ is the modulation applied to the signal. Inside of the lock-in amplifier, the input signal is mixed with a sinusoidal reference voltage at frequency $\omega_{0}$ and with a phase $\phi_{r}$ , which combines to give a mixed trace of

(4) $$\begin{array}{cc} \;z(t)= & v_{r}\left(t\right)v\left(t\right)\\ = & a\left(t\right)\sin\left(\omega_{0}t+\phi_{r}\right)\sin\left(\omega_{0}t+\phi_{s}\right)+b\left(t\right)\sin\left(\omega_{0}t+\phi_{r}\right),\\ = & \frac{a(t)}{2}\{\cos\left[\left(\left(\phi_{r}-\phi_{s}\right)\right]-\cos\left[2\omega_{0}t+\left(\phi_{r}+\phi_{s}\right)\right]\right\} \end{array}$$

(5) $$\begin{array}{cc} \; & +b\left(t\right)\sin\left(\omega_{0}t+\phi_{r}\right), \end{array}$$

which has a “pseudo” DC component (assuming that a(t) varies much more slowly than $\omega_{0}$ ), a component at $2\omega_{0}$ , and a component at $\omega_{0}$ . After mixing, this signal is passed through a low-pass filter that suppresses the components at $\omega_{0}$ and $2\omega_{0}$ , while passing through the component $a\left(t\right)\cos[\left(\left(\phi_{r}-\phi_{s}\right)\right]$ . Typically, lock-in amplifiers have the option either of outputting both the amplitude a(t) and the relative phase $\phi_{r}-\phi_{s}$ , or the two complex components $x\left(t\right)=a\left(t\right)\sin\left(\phi_{r}-\phi_{s}\right)$ and $y\left(t\right)=a\left(t\right)\cos\left(\phi_{r}-\phi_{s}\right)$ , with x(t) being the in-phase component and y(t) being the quadrature component.

#### 2.1.1. Modeling Background Suppression

To model background suppression using a lock-in amplifier, we assume that a(t)=0 and $\phi_{s}=\phi_{r}=0$ , such that v(t)=b(t) and the mixed signal is

(6) $$\begin{array}{c} z\left(t\right)=\frac{1}{2i}b\left(t\right)\left[e^{i\omega_{0}t}-e^{-i\omega_{0}t}\right] \end{array}$$

where we have written the sine function in complex form. To apply the lock-in amplifier’s low-pass filter, we first have to Fourier Transform z(t) into frequency space, which is as follows:

(7) $$\begin{array}{cc} Z(\omega ) & =\frac{1}{2i}\left[F\left\{b\left(t\right)e^{i\omega_{0}t}\right\}-F\left\{b\left(t\right)e^{-i\omega_{0}t}\right\}\right]\\ \; & =\frac{1}{2i}\left[B\left(\omega \right)*\delta \left(\omega -\omega_{0}\right)-B\left(\omega \right)*\delta \left(\omega +\omega_{0}\right)\right]\\ \; & =\frac{1}{2i}\left[B\left(\omega -\omega_{0}\right)-B\left(\omega +\omega_{0}\right)\right] \end{array}$$

where we have made use of the Convolution Theorem (with ∗ denoting convolution), recalling that $F\left\{e^{i\omega_{0}t}\right\}=\delta \left(\omega -\omega_{0}\right)$ .

Having determined Z(ω) , we apply the long-pass filter by multiplication (i.e., $Z\left(\omega \right)X_{n}\left(\omega \right)$ ), where the filter transfer function is

(8) $$\begin{array}{cc} X_{n}\left(\omega \right) & =X_{1}^{n} \end{array}$$

(9) $$\begin{array}{cc} \; & =\frac{1}{{\left(1-i\omega /\omega_{c}\right)}^{n}}. \end{array}$$

with $X_{1}\left(\omega \right)$ being the transfer function for a single long-pass filter, $\omega_{c}=1/\left(2\pi \tau \right)$ , where τ is the filter time constant, and *n* is the order of the filter, which determines the roll-off of the filter as 6n dB/octave. After applying the filter transfer function, we then take the inverse Fourier Transform to obtain the complex output of the lock-in amplifier:

(10) $$\begin{array}{cc} \;v_{out}\left(t\right) & =F^{-1}\left\{Z\left(\omega \right)X_{n}\left(\omega \right)\right\}\\ \; & =\frac{1}{2i}\left[F^{-1}\left\{B\left(\omega -\omega_{0}\right)X_{n}\left(\omega \right)\right\}-F^{-1}\left\{B\left(\omega +\omega_{0}\right)X_{n}\left(\omega \right)\right\},\right]\\ \; & =\left[b\left(t\right)\sin\left(\omega_{0}t\right)\right]*\left[\chi_{n}\left(t\right)\right], \end{array}$$

(11) $$\begin{array}{cc} \; & =\int_{0}^{\infty }b\left(\tau \right)\sin\left(\omega_{0}\tau \right)\chi_{n}\left(t-\tau \right)d\tau \end{array}$$

where we once again have made use of the convolution theorem. Note that the inverse Fourier transform of the transfer function $X_{n}\left(\omega \right)$ is given by

(12) $$\begin{array}{c} \chi_{n}\left(t\right)=2\sqrt{\frac{\pi }{2}}\frac{\omega_{c}^{n}}{\Gamma (n)}t^{n-1}e^{-\omega_{c}t}\Theta \left(t\right) \end{array}$$

where Γ is the Gamma function and Θ(t) is the Heaviside step function which is 0 for t≤0 and 1 for t>0 . Therefore, the integration range of Equation (11) is only [0,∞) as the integrand is 0 for t≤0 .

For this study we modeled (and measured) $v_{out}\left(t\right)$ for a Super-Gaussian background pulse given by

(13) $$\begin{array}{c} b_{\beta }\left(t\right)=b_{0}\exp\left\{-{\left(\frac{t-t_{0}}{W}\right)}^{\beta }\right\}, \end{array}$$

where $b_{0}$ is the amplitude, $t_{0}$ is the location of the peak center, *W* is the pulse width, and β is the power. Note that we chose this pulse shape as it allows us to easily test the effects of pulse steepness and width. It does not necessarilly correlate to the light curves of any specific explosive, whose light curves can vary based on composition and geometry [37].

We performed this modeling as a function of multiple different parameters, including reference frequency, lock-in time constant, lock-in roll-off, β parameter, and pulse width *W* to obtain the complex $v_{out}\left(t\right)$ for each configuration of parameters. After determining the complex $v_{out}\left(t\right)$ , we next computed its peak magnitude *R_p_*, from which we computed the background suppression in dB as

(14) $$S=-10\log\left(\frac{R_{p}}{b_{0}}\right).$$

#### 2.1.2. Super-Gaussian Fourier Transforms

As the modeling and experimental pulses of this study follow Equation (13), it is worthwhile to sketch out the functional form of their Fourier Transforms as this functionality helps us to understand the results we observe. We begin by noting that there is no simple analytical solution to the Fourier transform for all β and that we will only be considering even β ’s. For the simplest case of β=2 (or a normal Gaussian), the Fourier Transform is

(15) $$\begin{array}{c} B_{2}\left(\omega \right)=e^{-\frac{1}{4}\omega^{2}W^{2}} \end{array}$$

which is a well-known result. Turning to β>2 , we find a significantly more complicated functionality given by

(16) Bβ(ω)=∑jb−1ajωj,0Fβ−20,{cj};ωβwβββ

where $a_{j}$ are expansion coefficients, ,0Fb−2() are the generalized hypergeometric functions with zero *a* coefficients, and $\left\{c_{j}\right\}$ is a set of β−2 coefficients for the hypergeometric function. Note that the values of $a_{j}$ and $\left\{c_{j}\right\}$ change for each β value. For the purposes of this paper, we will not go into the detailed forms of these coefficients, as the important point of Equation (16) is that the Fourier spectrum of these functions depends on a series of hypergeometric functions which are oscillatory in both the frequency and width. This oscillatory behavior results in multiple zeros occurring for different combinations of ω ’s and *W*’s. These zeros lead to the observation of dips in the suppression curves for certain combinations of frequency and pulse width that have nothing to do with the lock-in functionality, but are instead due to dips in Fourier spectrum of the background pulse. This will be discussed further in the results section below.

#### 2.1.3. Temperature Modeling

In addition to modeling the lock-in amplifiers effects on background light, we also perform modeling on the impact of the lock-in amplifier technique on temperature profiles measured by two-color fluorescence thermometry. To begin, we assume a simplified lump-capacitance heating model of the thermographic phosphor, with its temperature in response to a hot gas being given by the differential equation:

(17) $$\begin{array}{c} \frac{dT}{dt}=\left(T_{g}\left(t\right)-T\left(t\right)\right)/\tau , \end{array}$$

where $T_{g}$ is the time-dependent gas temperature and τ is the characteristic time constant, which we calculate to be 325 μs based on previous modeling [36]. Note that the lumped-capacitance model is appropriate as the estimated Biot number for our fiber-based sensors is $3\times 10^{-3}$ .

To model the influence of the phosphor’s temperature on the fluorescence, we utilize a simple two-level population model where the excited state is split into two levels, with their populations being given by the Boltzmann distribution. Mathematically, this corresponds to a system of two coupled differential equations:

(18) $$\begin{array}{c} \frac{dn_{g}}{dt}=-\alpha I\left(t\right)n_{g}+\beta n_{e}, \end{array}$$

(19) $$\begin{array}{c} \frac{dn_{e}}{dt}=\alpha I\left(t\right)n_{g}-\beta n_{e}, \end{array}$$

where I(t) is the time-dependent intensity, α and β are rate constants, $n_{g}$ is the population in the ground state, and $n_{e}$ is the population in the excited state which is split into two sub-levels $n_{1}$ and $n_{2}$ , whose ratio is given by $e^{-\Delta E/kT(t)}$ . Note that in this model we make several assumptions for simplicity:

1. Instantaneous thermalization of the excited state levels.
2. Both excited states have the same decay rate β .
3. The rate constants α and β are invariant with temperature.

Assumption 1 is valid, as the thermalization timescale is on the order of picoseconds to nanoseconds [46], while assumptions 2 and 3 are less valid. Namely, the rate constants are known to vary with temperature, which is the basis of lifetime-based temperature sensing [46]. However, for Dy:YAG, both lifetimes are found to be relatively constant up to 1200 K—only decreasing by ≈ 10% at 1400 K—and within uncertainty of each other for the entire temperature range [17]. Additionally, while the change in the lifetime will impact the lock-in signal strength, it does so proportionally for both signals, such that the ratio should be unchanged. While this model may not accurately predict exact experimental data (depending on the thermographic phosphor modeled), it is sufficient to understand the impact of the lock-in amplifier on temperature measurement results, which is our primary goal with this modeling.

Practically, modeling begins by first solving Equation (17) for the phosphor’s temperature curve T(t) . Once this is determined, Equations (18) and (19) are solved assuming a laser pulse train with a repetition rate $\omega_{0}$ to obtain both $n_{g}$ and $n_{e}$ . Once $n_{e}\left(t\right)$ is determined, we split it into the two populations $n_{1}$ and $n_{2}$ using the Boltzmann’s distribution and the temperature curve determined earlier. For simplicity, we assume a one-to-one relationship between the fluorescence intensity and population such that the intensity profiles going into the lock-in amplifier are equal to the populations of $n_{2}$ and $n_{1}$ . After we pass the “intensities” through the model lock-in amplifier, we calculate their time-dependent ratio from which we determine our temperature trace using T(t)=−ΔE/(kln[r(t)]) , where r(t) is the time-dependent ratio.

Note that in addition to modeling the phosphor’s fluorescence, we also include a two-level model of fluorescence from the silica fiber using a lifetime of 0.5 μs, which we determined experimentally. This fluorescence is included in the model to simulate phase-locked detection where the lock-in amplifiers phase is set such that the fiber fluorescence is wholly in the quadrature component, while the phosphor’s fluorescence is primarily in the in-phase component. This silica fiber fluorescence modeling is primarily used in Section 3.1, where we consider the signal strength as a function of repetition rate.

### 2.2. Experimental

The experimental setup for measurements in this study is based on our previously reported optical thermocouple (OTC) system [36,37]. This system consists of a custom-built fiber probe, long silica coupling fibers, an optical analysis system, and a pump laser. The fiber probe is comprised of a 425 μm sapphire fiber with one end coated with Dy:YAG by pulse laser deposition. Details of this deposition process can be found in reference [36]. For the probes used in this study, the films were comprised of a 2 mol% concentration Dy:YAG with a thickness of approximately 2 μm and an average fluorescence lifetime of 660 μs. Note that this lifetime is less than our previous probes which used a 1 mol% concentration, with this reduction in lifetime a well-known phenomenon [47].

**Figure 1:**
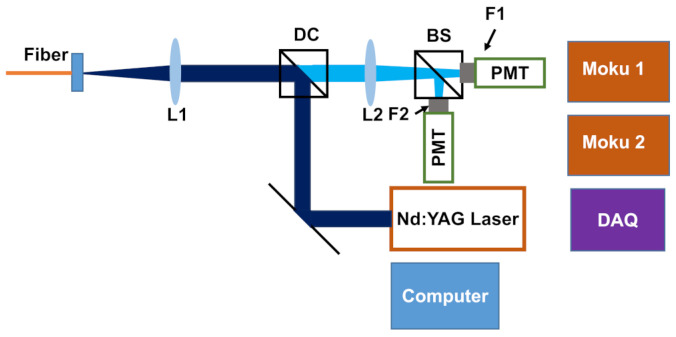
Schematic of optical setup used in this study.

For connecting the fiber probe to the analysis system, we used 5 m of 400 μm core, High-OH, solarization resistant fiber (Thorlabs (Newton, NJ, USA) FG400AEA) with FC/PC connectors on both the silica and sapphire probe. The coupling fiber connected to an optical analysis system, shown schematically in Figure 1, which coupled pump light from a Photonics Industries (New York, NY, USA) DCH355 laser (355 nm, 0–200 kHz) into the coupling fiber and collimated the returning fluorescence for analysis by two band-pass-filtered PMTs (Thorlabs (Newton, NJ, USA) PMT1001, 0.57 ns rise time (614 MHz bandwidth), 10 nA dark current (0.1 mV)). For band-pass filters, we used Edmund Optics (Newton, NJ, USA) # 65-142 (458 nm center, 10 nm bandwidth, OD4) and #65-149 (500 nm center, 10 nm bandwidth, OD4). Note that we used the the minimum gain setting for the PMTs such that the raw PMT signals were well below saturation, but were three orders of magnitude larger than the dark current. In this regime, shot noise dominates the experimental uncertainty and there are no effects due to saturation.

In our previous work, the outputs from the PMTs were directly connected to a DAQ device. However, for this study, we connected each PMT to its own Liquid Instruments (Solana Beach, CA, USA) Moku:Lab multi-instrument device (running in lock-in mode) with the two lock-in outputs sent to an National Instruments (Austin, TX, USA) USB DAQ (USB-6356). The DAQ was also used to measure the raw signals from each PMT and the laser sync reference. Note that this reference signal was used to perform phase-locking with the lock-in amplifier during our signal intensity measurements (Section 3.1). This phase-locking was performed by measuring the *X* and *Y* components from the lock-in amplifer using fluoresece from our silica coupling fiber (without an attached Dy:YAG probe). Using this data, we determined the phase necessary to put all of the silica fiber fluorescence in the *Y* component. This ensured that when we attached the probe, the signal in the *X* component would only be due to the Dy:YAG. This phase-locking approach was necessary for the signal strength measurements as the quantities we measured are phase-dependent. However, for the background suppression measurements, we computed suppression using the lock-in signal amplitude, which is independent of phase. Note that the other lock-in parameters (e.g., frequency, time constant, and roll-off) for each measurement are all listed in the results section below.

Finally, we introduced background light pulses into the fiber probes using a Thorlabs (New Jersey, USA) Solis 3C High-Power LED (white light 5700 K, 3.5 W, <1% pulse-to-pulse intensity variation) pointed at the fiber tip. We controlled the pulse shape of the LED light using a Thorlabs (New Jersey, USA) DC2200 LED driver using external modulation coming from a Moku:Lab device running as an arbitrary waveform generator, where the pulse shape is defined by Equation (13). To account for experimental uncertainties/variations in the LED light, PMTs, and lock-in amplifier, we performed each background suppression measurement five times and computed the resulting average suppression and its uncertainty. In general, the uncertainties in the background suppression values are <3%.

## 3. Results

### 3.1. Effect of Laser Repetition Rate

#### 3.1.1. Signal Strength

To begin our study of the effects of different experimental parameters on lock-in-based OTC, we first consider the influence of laser repetition rate on both the fluorescence signal and background suppression. From our experiments, we find that the measured fluorescence signal consists of a short lifetime component coming from silica fiber fluorescence and the much longer lifetime Dy:YAG fluorescence. To separate out these components using the lock-in, we first measure the signal from the fiber alone (without a probe attached) and adjust the lock-in’s phase until all of the fiber’s fluorescence is in the quadrature component. With these phase settings, the in-phase component of the full assembly will come only from the Dy:YAG.

**Figure 2:**
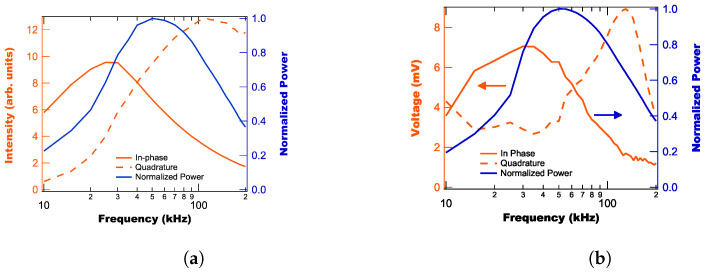
Modeled (**a**) and experimentally measured (**b**) Lock-in amplifier signal intensities as a function of voltage.

Using this method of phase separation, we both modeled and experimentally measured the in-phase and quadrature signal as a function of laser repetition rate (using a lock-in time constant of 1 ms and a roll-off of 18 dB/oct.), with the model results shown in Figure 2a and the experimental results shown in Figure 2b. Note that we also plot the laser’s power as a function of repetition rate in Figure 2 for reference as the laser’s power is not constant with frequency. This effect is included in the model results.

From Figure 2, we find that while there are some differences in the model results and experimental data, the in-phase curves are consistent between both. The primary point of disagreement between model and experiment is with regards to the quadrature component which is related to the silica fiber fluorescence, whose model was ad-hoc. Additionally, from Figure 2, we find that both the in-phase and quadrature components behave differently with laser frequency and that neither follows the power curve. For instance, the peak laser power occurs at 50 kHz, while the peak in-phase signal is at 35 kHz and the quadrature signal peaks above 100 kHz. This difference in peak location is related to how the different material’s fluorescence lifetimes impact their signals.

To understand how this effect works, we highlight that the average lifetime of our probes is ≈ 660 μs. This means that the fluorescence decay will only have reached 65% in between pulses for the 35 kHz frequency (286 μs period), which is a significant reduction in the amplitude of oscillation, which only gets worse as the frequency increases. Therefore, while the DC component of the fluorescence signal peaks with the laser power, the amplitude of oscillation does not. Since the lock-in amplifier measures this amplitude and removes the DC offset, the in-phase and quadrature signals end up peaking at frequencies related to their respective lifetimes.

**Figure 3:**
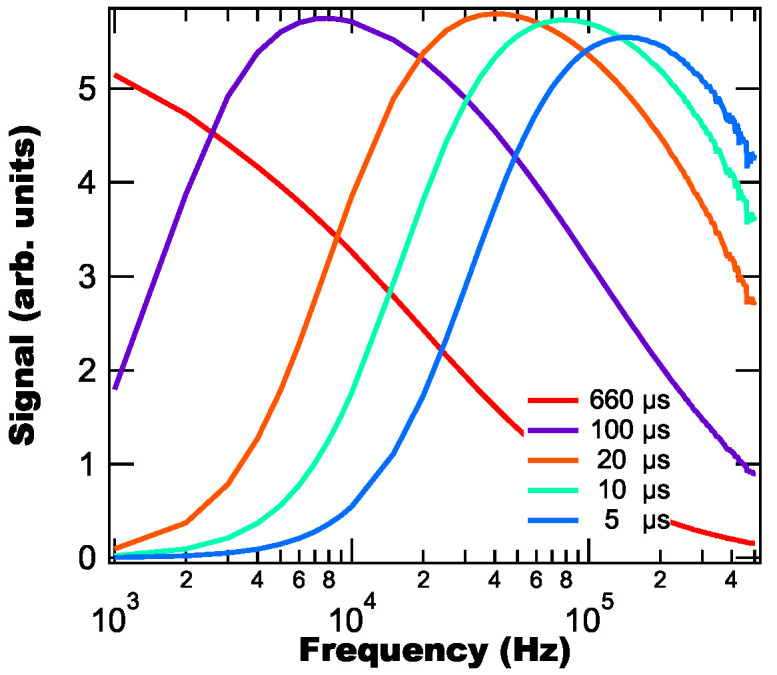
Modeled lock-in signals as a function of frequency for different fluorescent lifetimes for a constant average laser power.

This relationship between the peak lock-in signal and the fluorescence lifetime has important implications for using the lock-in approach for other thermographic phosphors (e.g., Dy:YAG, Eu:BAM, Dy/Sm-based organic complexes, Dy/Eu-doped crystalline hosts) which all have unique lifetimes. To demonstrate this effect, we model a lock-in’s signal intensity as a function of frequency using fluorescence pulse trains with different lifetimes—and assuming the average power is constant as a function of frequency—with the resulting signals as a function of frequency shown in Figure 3. From Figure 3 we find that the signal peak shifts towards higher frequencies as the lifetimes decreases and that the peak frequency is slightly less than the inverse lifetime (e.g., the 100 μs lifetime peaks at 8 kHz, which is less than the inverse lifetime of 10 kHz).

#### 3.1.2. Background Suppression

Having considered how the lock-in signals change with repetition rate, we now turn to considering how the frequency impacts the lock-in’s background suppression. Note that for the sections on background suppression, we switch from considering the in-phase and quadrature components of the lock-in signal and instead use the lock-in amplitude to define suppression.

**Figure 4:**
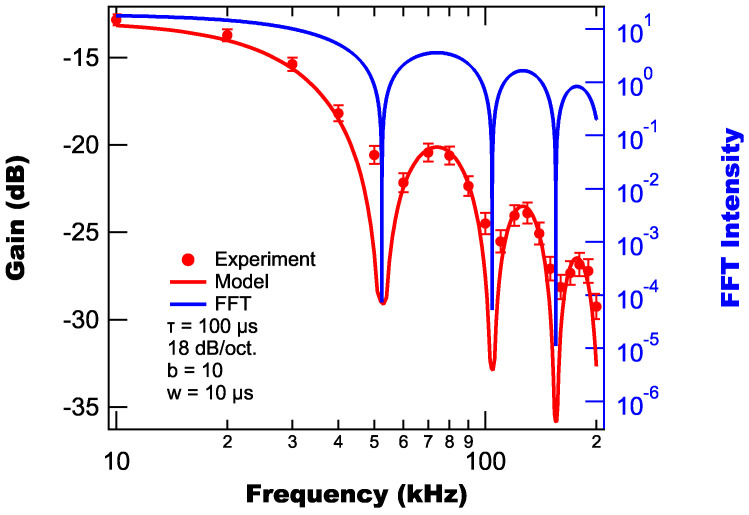
Modeled (line) and experimentally measured (markers) background suppressions as a function of laser repetition rate.

As mentioned above, we both model and experimentally measure light pulses defined by Equation (13) for different combinations of experimental parameters. For characterizing the influence of reference frequency on the suppression, we use a parameter set of β=10 , W=10 μs, 18 dB/oct., and τ=100 μs, with the resulting suppressions shown in Figure 4. From Figure 4, we find that the background suppression increases as the reference frequency increases with several dips observed in the suppression curves at 55 kHz, 110 kHz, and 165 kHz. These dips coincide with dips in the light pulses’ FFT spectrum (see Figure 4) and therefore are due to the light pulses’ structure and not the function of the lock-in amplifier.

Additionally, we find from Figure 4, that while the majority of experimental data points agree with the model (within uncertainty), there are several that deviate outside of uncertainty, primarily near the dips. Most likely, these deviations are due to imperfections and noise in the experimental light pulses, which leads to variations in the Fourier spectrum of the light pulses. These variations can lead to uncertainty in the dip locations and their widths, which when averaged over multiple experimental runs, results in the dips not being perfectly reproduced. These issues are less pronounced away from the dips as the spectrum is less sensitive away from the dips.

### 3.2. Lock-in Time Constant

**Figure 5:**
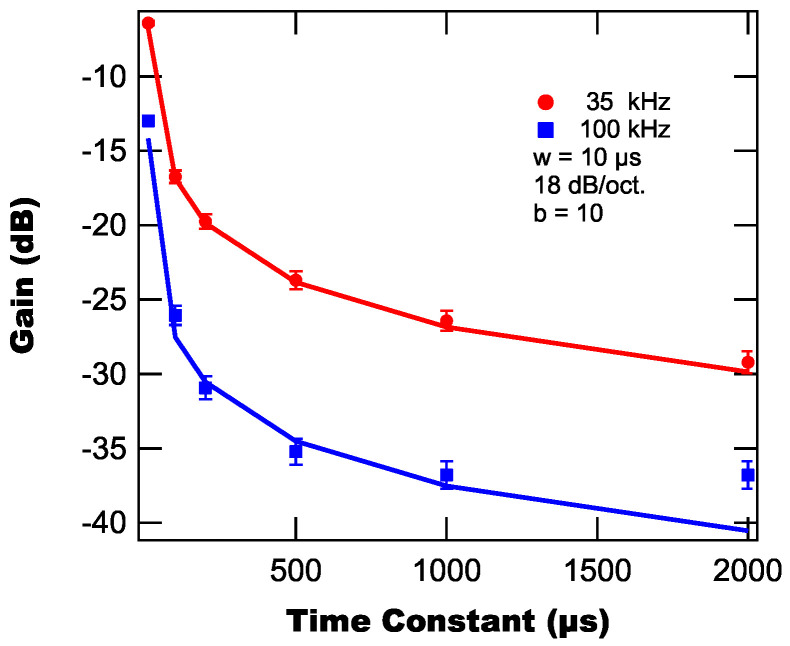
Modeled (lines) and experimentally measured (markers) background suppressions as a function of lock-in time constant.

After characterizing the effect of the reference frequency on background suppression, we next turn to the influence of the lock-in time constant with a parameter set of W=10 μs, β=10 , 18 dB/oct., and two frequencies (35 kHz and 100 kHz). Note that we consider two different frequencies as we found above that the peak signal occurs at 35 kHz, but also that the suppression increases with frequency. Therefore, we chose to test an additional higher frequency (100 kHz), where the signal is still adequate but the suppression will be superior. Figure 5 shows both the modeled and experimentally measured suppressions as a function of lock-in time constant. From Figure 5, we find that the suppression follows a sub-linear power function decay as the time constant is increased and that the suppression is greater for 100 kHz than for 35 kHz.

The increase in suppression as the time constant increases is to be expected as the time constant determines the corner frequency of the low-pass filter. By increasing the time constant, the corner frequency decreases and the suppression on higher frequency components increases. This provides improved suppression of short-duration background pulses, which is advantageous for explosive fireballs. However, this improved suppression comes at a cost as the same filter that suppresses the unwanted high frequency background also suppresses the high frequency components in the wanted signal. Practically, this means that the thermal response of the OTC is smeared out in time by the lock-in amplifier with response time being limited by the lock-in’s time constant.

**Figure 6:**
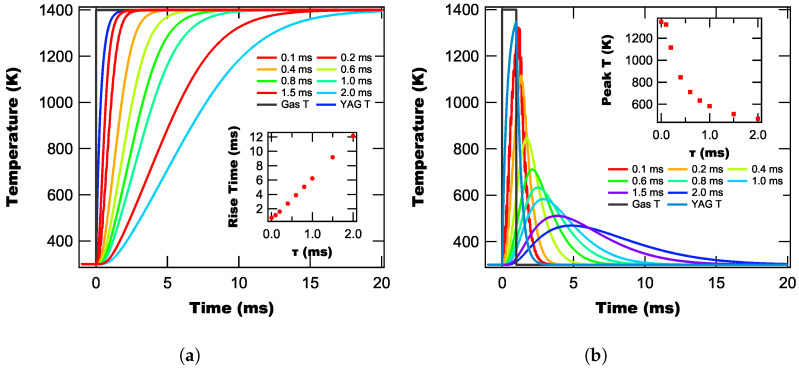
Modeled temperature curves for the lock-in based OTC method in response to a step temperature curve (**a**) and a 1 ms box pulse (**b**) for different lock-in time constants.

To better understand the impact of the lock-in’s time constant on the OTC’s measured temperature curves, we model the OTC’s response to both a temperature step (Figure 6a) and a 1 ms duration temperature pulse (Figure 6b) for different time constants. Once the temperature curves were modeled, we then computed their rise times (defined as reaching 90% peak temperature) for the step temperature profile; and for the pulse profiles, we computed the peak calculated temperature for each time constant, with each of these quantities plotted in the relevant insets in Figure 6. Note that we performed this modeling for both 35 kHz and 100 kHz and find that the results are invariant with reference frequency.

From Figure 6a, we find that the temperature calculated by the lock-in technique in response to a temperature step is slower than for the YAG film itself, even when the lock-in’s time constant is lower than the YAG’s rise time. This means that regardless of the time constant, the temperature calculated by the lock-in will always lag the actual temperature of the YAG film. We also find from the inset in Figure 6a that the rise time increases linearly with the time constant, with the rise time being approximately 6× the time constant for 18 dB/oct.

Turning to the model results in response to a square peak (Figure 6b), we once again find that the response time using the lock-in is slower than the film’s actual temperature response, that the peak width increases with time constant, and that the peak temperature measured by the lock-in method is lower than the actual peak temperature of the YAG film. From the inset in Figure 6b, we find that the measured peak temperature decays exponentially with the time constant, which means that at the lower end of time constants, small increases produce significant degradation in performance. However, as the time constant continues to increase, the decline in performance levels out.

Practically, what these results mean is that for fast heating events (e.g., explosions), we want to use the smallest time constant possible to minimize the impact of the lock-in on our measured temperature curves. However, from our background suppression measurements, we know that longer time constants are better for background suppression. Therefore, there is a trade-off between background suppression and temperature response accuracy that needs to be taken into account when choosing lock-in parameters for a given experiment. This will be discussed later on.

### 3.3. Lock-in Roll-Off

**Figure 7:**
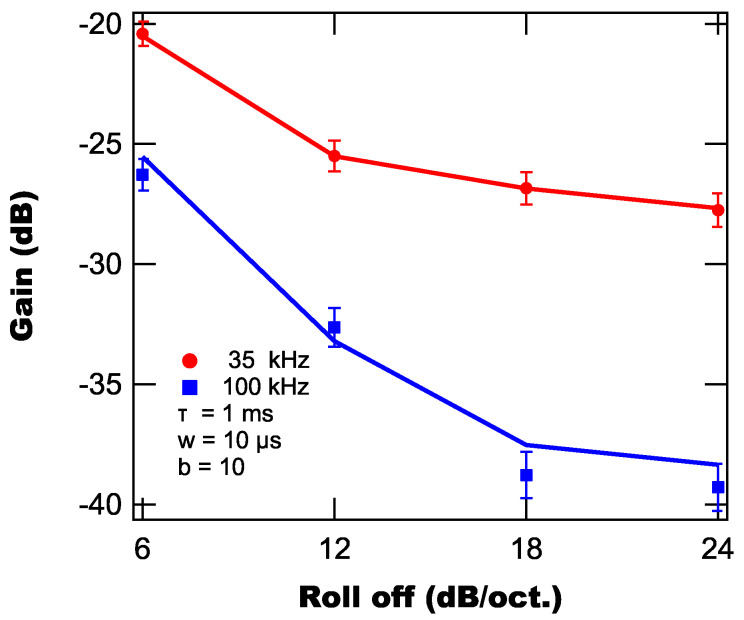
Modeled (lines) and experimentally measured (markers) background suppressions as a function of lock-in roll off.

The second lock-in parameter that affects the time response of the lock-in is the long-pass filter’s roll-off, which is proportional to the filter’s order. The roll-off is a measure of the filter’s steepness (i.e., how quickly the filter’s suppression increases as the frequency increases) with each additional filter order adding another 6 dB/oct. of steepness. This increase in steepness improves the filter’s suppression of higher frequency components, helping improve the suppression of background light. To demonstrate this effect, we once again modeled and measured the lock-in’s response to our test light pulse for a time constant of 1 ms, β=10 , W=10 μs, and both 35 kHz and 100 kHz, with the results shown in Figure 7.

From Figure 7, we find similar functional behavior between the model and experimental data with the suppression decreasing roughly linearly with roll-off until 18 db/oct. after which the improvement in suppression drastically slows. Practically, this means that using a roll-off above 18 dB/oct does not provide any significant benefit over the 18 dB/oct setting, but comes with negative impacts on the time response of the temperature sensor, as will be described next.

**Figure 8:**
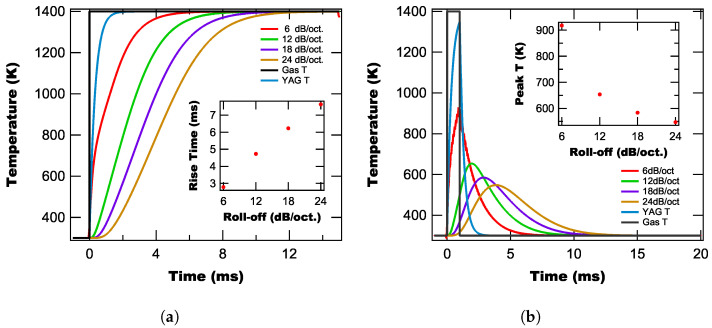
Modeled temperature curves for the lock-in based OTC method in response to a step temperature curve (**a**) and a 1 ms box pulse (**b**) for different lock-in time constants.

Turning to the impact of roll-off on the OTC’s temperature measurement, we model the system’s response to a temperature step function and a square temperature pulse as above, with the resulting lock-in calculated temperatures shown in Figure 8. We additionally include the gas and YAG temperature curves for reference. From Figure 8, we find that the roll-off has a similar impact on the temperature curves as the time constant, with the rise time increasing linearly with roll-off (see the inset in Figure 6a) and the peak temperature decreasing exponentially with roll-off (see the inset of Figure 6b). As with the time constant above, we find that while increasing the filter’s roll-off helps to improve suppression of background light, it comes at the cost of a slower time response of the OTC and a decrease in the peak temperature measured by the lock-in technique.

### 3.4. Time Constant, Roll-Off, and Temperature Pulse Width

At this point, we note that we have thus far only considered the OTC’s response to a 1400 K pulse with a width of 1 ms. While this modelling is sufficient to provide an initial understanding of how lock-in detection affects the OTC’s temperature measurement, it misses the covariance between the lock-in’s time constant, roll-off, and the width of the temperature pulse. To provide an exploration of this covariance, we also modeled the temperature response of the OTC for different permutations of variables for both the step function and pulse temperature profiles. We then computed the rise time for a step temperature profile and the peak temperature and Pearson correlation coefficient for the pulse temperature profile, where the correlation coefficient provides a measure of how closely the lock-in’s temperature trace reproduces the actual temperature trace.

**Figure 9:**
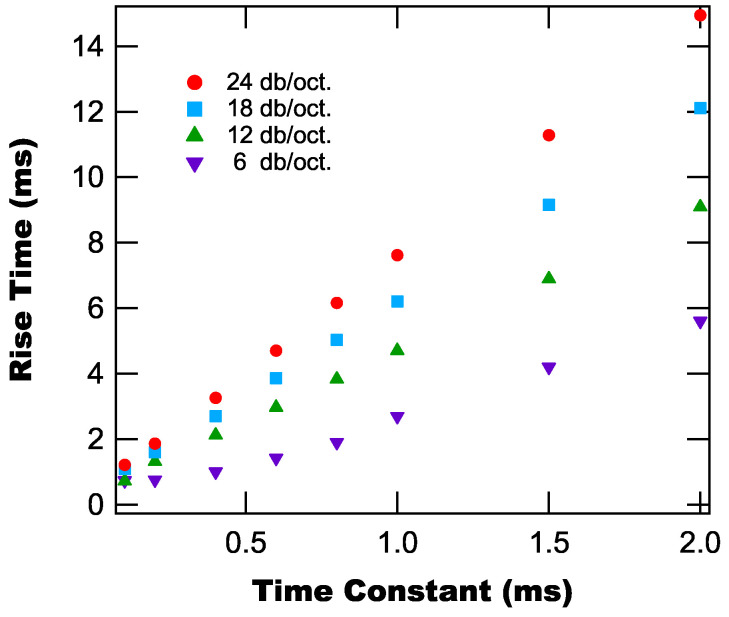
Rise time in response to a step function temperature as a function of time constant for different roll-off values.

Figure 9 shows the rise time as a function of time constant for different roll-off values. From Figure 9, we once again find that the rise time is linear with time constant, with the slope increasing with increasing roll-off. These results are a combination of the dependencies seen above, but demonstrate the explicit covariance of the rise time on both time constant and roll-off.

**Figure 10:**
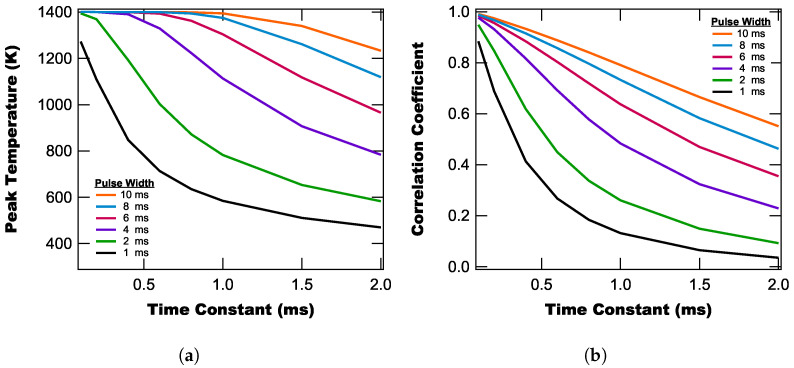
Peak temperature (**a**) and correlation coefficient (**b**) of the lock-in’s temperature curve as a function of time constant for different pulse widths with a roll off of 18 dB/oct.

We next consider the covariance of the pulse width and time constant (while holding the roll-off constant at 18 dB/oct.) by modeling temperature pulses with different pulse widths and a peak temperature of 1400 K. From these modeled temperature profiles, we calculate the peak temperature, shown in Figure 10a, and their correlation coefficient (relative to the actual temperature profile), which is shown in Figure 10b.

From Figure 10, we find that the peak temperature and correlation coefficient both decrease as the time constant increases. However, we also observe that the exact functionality depends on the pulse width, with pulses having larger widths being better reproduced by the lock-in temperature traces. We also note that based on the curves in Figure 10, the actual peak temperature (1400 K) is reproduced as long as the pulse width is approximately 10× longer than the time constant. This means that when using the lock-in approach for slower heating events, we would anticipate better accuracy than when using it for fast heating events.

### 3.5. Pulse Width

**Figure 11:**
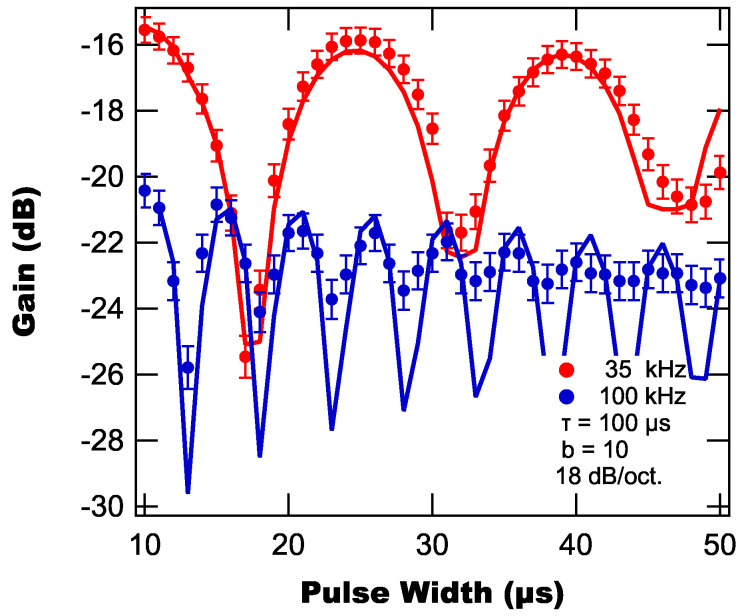
Modeled (lines) and experimentally measured (markers) background suppressions as a function of pulse width.

Thus far, we have considered how different lock-in parameters affect the background suppression and temperature measurement of the OTC. At this point, we turn to considering how the shape of the background pulse affects suppression, with our test pulse (Equation (13)) having two shape parameters *W* and β , with *W* being the pulse width and β being an exponential parameter that effects the steepness of the pulse. The first parameter that we consider is the pulse width, with Figure 11 showing the measured and modeled suppression for both 35 kHz and 100 kHz using a time constant of 100 μs, β=10 , and a roll-off of 18 dB/oct.

From Figure 11, we find that the background suppression displays oscillatory behavior as a function of pulse width with the period of oscillation depending on the reference frequency. This behavior is a consequence of the background pulse’s Fourier spectrum (see Equation (16)) containing hypergeometric functions of the product ${\left(\omega W\right)}^{\beta }$ , which has zeros corresponding to different combinations of ω and *W*. This dependence on the width in the Fourier spectra also explains why there are deviations between model and experiment. For the model, the light pulses are ideal with no noise nor any uncertainty in the pulse width. However, in the experiment, there is noise and some uncertainty in the peak width, which results in the experimental Fourier spectrum varying slightly from pulse to pulse, which leads to deviations from the model. Note that while there are these oscillations, the overall trend of the suppression is a slight increase as the pulse width increases. However, this increases only corresponds to a few dB, which is minor compared to the effect of the lock-in parameters considered above.

### 3.6. β Parameter

**Figure 12:**
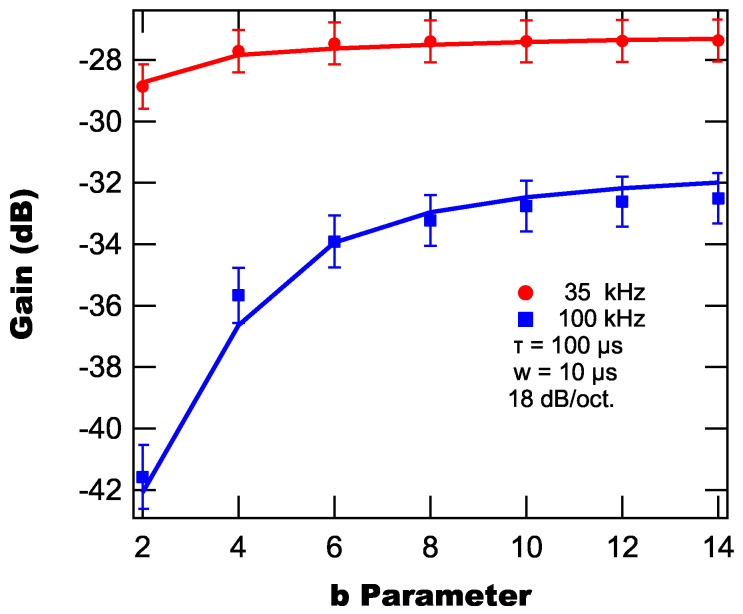
Modeled (lines) and experimentally measured (markers) background suppressions as a function of the pulse parameter *b*.

The next pulse shape parameter we consider is β , which influences the sharpness of the peak and can be understood as a surrogate parameter for a generalized background light’s rise time. The larger β is, the faster the background light pulse increases, which allows us to characterize the lock-in response to different rates of increasing intensity. Figure 12 shows the modeled and measured background suppression as a function of β parameter for a time constant of 100 μs, a pulse width of 10 μs, a roll-off of 18 dB/oct. and reference frequencies of 35 kHz and 100 kHz.

From Figure 12, we find that the suppression decreases as the *b* parameter increases and that the suppression eventually plateaus as a function of *b*, with the onset of this plateau occurring at higher *b* values as the reference frequency increases. Practically, this means that the faster the background light rises, the less effective the lock-in amplifier is at suppressing its signal.

### 3.7. Temperature Uncertainty Estimates

Thus far, we have considered the effect of lock-in detection on background suppression (using both modeling and experiment) and the effect of these parameters on modeled temperature calculations. At this point we now turn to providing uncertainty quantification of the temperature calculation using the lock-in detection method. To begin, we will consider the steady state or slowly evolving temperature uncertainty, as the uncertainty for a transient temperature pulse depends on the structure of the temperature pulse.

To calculate the “steady-state”’ temperature uncertainty, we recall that the experimentally measured intensity ratio follows an exponential function [36]:

(20) $$\begin{array}{c} r=r_{0}+Ae^{-T_{0}/T} \end{array}$$

where $r_{0}$ is a ratio offset, *A* is an amplitude parameter, and $T_{0}$ is the two-color phosphor’s characteristic temperature. Note that both $r_{0}$ and *A* depend on the experimental parameters as well as the phosphor properties, while $T_{0}$ depends only on the phosphor properties [48]. Rearranging Equation (20), we can write the temperature as a function of measured ratio as

(21) $$\begin{array}{c} T=\frac{-T_{0}}{\ln[\frac{r-r_{0}}{A}]}, \end{array}$$

from which we determine the uncertainty in our calculated temperature using error propagation to be as follows:

(22) $$\begin{array}{c} \sigma_{T}=\frac{T}{T_{0}}\sqrt{\sigma_{T_{0}}^{2}+T^{2}\left(\frac{\sigma_{A}^{2}}{A^{2}}+\frac{\sigma_{r}^{2}+\sigma_{r_{0}}^{2}}{{\left(r-r_{0}\right)}^{2}}\right)} \end{array}$$

where $\sigma_{i}$ are the uncertainties in the *i*th parameter. For our current Dy:YAG probes and experimental setup, we have measured our calibration parameters to be $T_{0}=1647\;\pm \;13$ K, $r_{0}=0.0669\;\pm \;0.0046$ , and A=8.984 ± 0.087 . Knowing Equation (22), the calibration parameters, and their uncertainties, the only remaining piece of information to determine the temperature uncertainty is the uncertainty in the measured ratio.

The uncertainty in the ratio is given by

(23) $$\begin{array}{c} \sigma_{r}=r\sqrt{{\left(\frac{\sigma_{I_{2}}}{I_{2}}\right)}^{2}+{\left(\frac{\sigma_{I_{1}}}{I_{1}}\right)}^{2}} \end{array}$$

where $I_{i}$ are the measured band-integrated intensities and $\sigma_{i}$ are their associated uncertainties. In general, the uncertainty in the intensities include dark noise, 1/*f* noise, digitization noise, and shot noise. However, in practice, we find that the majority of noise comes from shot noise and therefore, we estimate the intensity noise to be $\sigma_{I_{i}}=\sqrt{\sigma_{I_{i}}}$ .

**Figure 13:**
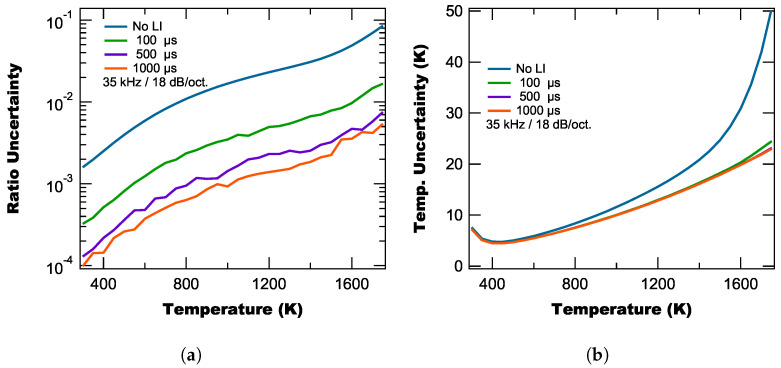
Calculated ratio (**a**) and temperature (**b**) uncertainty as a function of temperature for no lock-in amplifier and for three different lock-in time constants.

As shot noise is “white noise” (i.e., has a uniform frequency distribution), the effect of using a lock-in amplifier is to suppress the frequency components above the cut-off bandwidth, which results in the lock-in amplifier reducing the uncertainty in the measured intensities and therefore the measured ratio. Figure 13a shows the calculated ratio uncertainties as a function of temperature for different lock-in time constants, a reference frequency of 35 kHz, and a roll-off of 18 dB/oct. From Figure 13b, we find that utilizing a lock-in amplifier reduces the shot noise by over an order of magnitude, depending on the settings, which essentially makes the measured ratio’s contribution to Equation (22) negligible when compared to the other uncertainty terms.

Figure 13b shows the temperature uncertainty determined using Equation (22) for both the no-lock-in amplifier approach and the lock-in amplifier approach with the lock-in amplifier approach essentially producing an uncertainty curve only due to the calibration parameter uncertainty as the lock-in amplifier minimizes the effect of the measured ratio’s uncertainty.

### 3.8. Summary and Application Notes

In the sections above, we presented the results of our theoretical and experimental study on two-color fluorescence thermometry using lock-in amplifiers for background suppression. At this point, we summarize these results in Table 1, highlighting how the different lock-in parameters affect both background suppression and the temperature measurement.

From Table 1, it is apparent that there are trade-offs to consider when choosing lock-in amplifier parameters for implementation with two-color fluorescence thermometry. Based on our results, we find that it is best to use higher reference frequencies to improve background suppression. However, we also find that this frequency is limited by the lifetime of the thermographic phosphor, as the lock-in amplifier signal begins to decrease with frequency once the frequency is greater than the reciprocal lifetime. Therefore, there needs to be a balance between increased background suppression and improved fluorescence signal from the lock-in amplifier.

Turning to the time constant and roll-off, we find that increasing both of these quantities significantly improves background suppression to a point, after which the improvement slows. We find that for the roll-off this occurs at 18 dB/oct, and for the time constant, the exact value depends on both the reference frequency and characteristics of the background light pulse. While these background suppression results suggest that we want to use larger time constants and roll-offs, we observe the opposite scenario when considering the temperature measurement, as increasing both quantities results in longer temperature rise times and less accurate reproductions of underlying temperature pulses. Therefore, when choosing what time constant and roll-off to use for a given experiment, care must be taken to optimize background suppression while limiting the impact on the actual temperature measurement, with precise values depending on the expected timescales of the background and temperature fluctuations. Note that we find a rule-of-thumb that the time constant should be 1/10th the anticipated temperature peak width in order to accurately reproduce the peak temperature.

To provide an example scenario of choosing lock-in parameters using these guidelines, we recall that during previous explosive field tests of our OTC system [37], we found that the initial temperature pulse of a non-metallized explosive fireball was approximately 10 ms in width and that the initial background light pulse was approximately 400 μs in width, with its intensity being +20 dB larger than the fluorescence signal (when using the 35 kHz rep rate). If we assume a target signal-to-background ratio of 100, then we need a total of −40 dB suppression from the lock-in amplifier to adequately suppress the background. To achieve this level of suppression for a reference frequency of 35 kHz, we find that we need a time constant >5 ms, at a roll-off of 18 dB/oct.

For these parameters, we predict the lock-in amplifier’s temperature trace to have a rise time of approximately 30 ms, a peak temperature that is approximately 45% the actual peak temperature, and a correlation between the lock-in’s temperature trace and the actual pulse of ∼0.2. These poor performance parameters mean that the accuracy of the system’s temperature trace is severely degraded. To improve the temperature performance, we can increase the repetition rate to 100 kHz (which allows us to use a shorter time constant), but with this increase, the phosphor signal falls by −5 dB, which means we now need a total suppression of −45 dB from the lock-in amplifier to obtain our target signal-to-background ratio. To obtain this level of suppression at 100 kHz, we use a time constant of 2 ms, which results in the temperature trace now having a rise time of 6 ms, a peak temperature that is approximately 80% the actual peak temperature, and a correlation between the lock-in’s temperature trace and the actual pulse of ∼0.58. While this is an improvement over the 35 kHz configuration, it is still a poor reproduction of the underlying temperature curve.

Coming at the problem from a different angle, we consider at what pulse width the temperature reproduction becomes sufficiently accurate, using the 100 kHz repetition rate, 18 dB/oct. roll-off, and 2 ms time constant. For this calculation, we define a minimum accuracy of having a correlation coefficient of 0.9. We then performed modeling for different pulse widths and find that for temperature pulses with widths >42 ms, we obtain correlation coefficients >0.9. This means that for heating events with widths >42 ms, we can expect to obtain reliable temperature curve reproductions using the lock-in based approach with the current OTC design.

At this point, we note that the above example is based on using Dy:YAG as our thermographic phosphor, which has a room temperature lifetime of 660 μs that limits us to using repetition rates of a few hundred kHz before the signal becomes too weak. However, we can use higher frequencies if we switch phosphors, with one of the best candidates being Eu:BAM, which has a room temperature lifetime of approximately 2 μs [49,50]. In theory, this phosphor can be excited at 1 MHz and still produce a good lock-in signal. For this reference frequency, we only need a time constant of 100 μs at a roll-off of 18 dB/oct. to obtain the necessary background suppression. These settings result in a temperature rise time of 0.6 ms, which allows us to accurately resolve temperature peaks as short as 1 ms. However, while Eu:BAM provides superior time resolution, it is only useful as a thermographic phosphor up to approximately 900 K, at which point it undergoes oxidation resulting in the divalent Eu ions converting into trivalent ions [51,52,53].

As a final comment on applying this technique to other phosphors, we provide a non-exhaustive tabulation in Table 2 of two-color fluorescence phosphors, their lifetimes, max temperatures, and estimates of both their maximum laser frequencies and the estimated time constant to obtain −40 dB suppression for an 18 dB/oct. roll-off. Note that these are estimates which assume a constant laser power as a function of repetition rate and therefore, care must be taken when applying these results to specific experimental configurations.

### 3.9. Comparison to Other Background Suppression Techniques

Having concluded our study on using lock-in amplifiers for background suppression in two-color fluorescence thermometry and provided guidance on parameter settings for different thermographic phosphors, we now turn to a brief comparison of the three main background suppression techniques: lock-in amplfiers, reference subtraction, and gated detection. To compare these techniques, we summarize their different advantages and disadvantages in Table 3. From Table 3, we note that the lock-in amplifier technique is the only one that is guaranteed to account for background light interference (assuming appropriate parameters can be chosen), while the gated detection technique requires the background light to be dim relative to the fluorescence and the reference measurement technique may or may not see the same background signal. However, despite the lock-in amplifier’s advantage in background suppression, it has disadvantages due to the lock-in parameters possibly impacting the measured temperature profile and the sensitivity of lock-in amplifiers to their environment, which makes field-deployment challenging.

## 4. Conclusions

Lock-in amplifiers are an invaluable signal processing tool that allows for the extraction of small signals from significant background noise. Despite their usefulness in a wide array of experiments, they have been underutilized in the field of two-color fluorescence thermometry. To bring more attention to this technique, we have performed a comprehensive theoretical and experimental study of lock-in amplifier-based detection in two-color fluorescence thermometry, with a focus on the impact of the different relevant parameters on both background suppression and temperature measurement accuracy. We have demonstrated that there is a trade-off between improving background suppression and obtaining accurate temperature profiles, which needs to be carefully balanced by choosing an appropriate thermographic phosphor and lock-in parameters based on the expected timescales of both the temperature curves and background light. Based on these observations, we have provided a brief guide for other researchers to use when setting up their own two-color fluorescence thermometry experiments using lock-in amplifiers.

## Figures and Tables

**Table 1:** Summary of effects of lock-in parameters on background suppression and temperature measurement.

Lock-in Parameter	Background Suppression	Temperature Measurement
**Frequency**	• Increases with frequency	• No impact on temperature rise time or peak temperature. • Effects signal strength in combination with phosphor lifetime
**Time Constant**	• Increases with time constant, but eventually reaches a point of diminishing returns.	• Rise time is linear in time constant, with the slope dependent on the roll-off. • Correct peak temperature is obtained as long as the pulse width is 10× greater than the time constant. • For shorter pulses the peak temperature decays exponentially with time constant. • Correlation coefficient between actual pulse and reproduced pulse decreases with time constant.
**Roll-off**	• Significantly increases with roll-off up to 18 dB/oct, after which the increase per step is reduced.	• Rise time is linear in roll off, with the slope dependent on the time constant. • Peak temperature decays exponentially with roll off.

**Table 2:** List of different two-color fluorescence phosphors reported in the literature, their room temperature lifetime, maximum temperature, and estimated max frequency and minimum time constant to obtain a −40 dB suppression from a lock-in amplifier.

	Lifetime (μs)	*T_max_* (K).	Est. *F_max_* (kHz)	Est. *τ* (μs)	Ref.
Dy:YAG	660	1700	105.31	1054	This study
Dy:YVO_4_	158	673	477.03	547	[54]
Dy:BaYF_5_	1300	800	15.51	1230	[55]
Dy:YAM	662	1000	104.68	1056	[12]
Dy:BSAS	900	1700	51.35	1157	[56]
Dy,Er:YAG	592	1650	129.08	1012	[15]
Dy:YAG(BN)	606	1650	123.78	1021	“
Dy,Er:YAG(BN)	676	1650	100.38	1063	”
Dy:YAP	600	1700	126.02	1017	“
Dy:YSZ	390	1200	236.28	840	”
Dy:YSZ(Ca)	370	1100	250.86	819	“
Dy,Er:YSZ(Ca)	328	1100	284.47	772	”
Dy:YSO	438	1500	204.66	888	“
Dy,Er:YSO	354	1500	263.17	801	”
Dy,Pr:YSO	335	1500	278.57	780	“
Dy:CASO	563	1400	140.78	992	”
Sm:Y_2_O_2_S	400	1425	229.31	850	[57]
Eu:Y_2_O_3_	1392	1273	11.78	1238	[31]
Eu:YAlO_3_	1500	1300	8.52	1245	[58]
Eu:Y_3_Al_5_O_12_	3000	1470	0.10	1263	[58]
Eu:BAM	2	900	1305.03	95	[50]
Eu:CaWO_4_	211	773	404.46	623	[59]
Ce:YAG	0.1	923	1343.54	85	[60]
Pr:YAG	190	1100	431.33	594	[17]
Tm:YAG	100	1700	587.36	447	“
Eu:Y_2_O_3_	1000	1300	38.06	1184	”

**Table 3:** Comparison of different background suppression techniques for two-color fluorescence thermometry with their different advantages and disadvantages.

	Gated Detection	Reference Measurement	Lock-in Amplifier
**Advantages**	Only requires a single probe, making it the simplest and cheapest method	In theory can handle background light interference significantly brighter than fluorescence signal.	With appropriate lock-in parameters it can account for background light interference significantly brighter than fluorescence signal.
	No impact on T profile Simple to ruggedize for field deployment	No impact on T profile Simple to ruggedize for field deployment	Only requires a single probe.
**Disadvantages**	Fails when background signal is bright on same time scale as fluorescence lifetime.	Requires two probes and detection systems increasing size, cost, and complexity.	Requires careful tuning of parameters to obtain sufficient background suppression.
		There is no grantee that the background seen by both probes are identical.	Choice of lock-in parameters impacts measured T profile
			Lock-in Amplifiers are sensitive to environment making field-deployment challenging.

## Data Availability

Data underlying the results presented in this paper are not publicly available at this time but may be obtained from the authors upon reasonable request.
